# Extrachromosomal Nucleolus-Like Compartmentalization by a Plasmid-Borne Ribosomal RNA Operon and Its Role in Nucleoid Compaction

**DOI:** 10.3389/fmicb.2018.01115

**Published:** 2018-06-05

**Authors:** Carmen Mata Martin, Zhe Sun, Yan Ning Zhou, Ding Jun Jin

**Affiliations:** Transcription Control Section, RNA Biology Laboratory, Center for Cancer Research, National Cancer Institute, National Institutes of Health, Frederick, MD, United States

**Keywords:** RNA polymerase, bacterial nucleolus-like, rRNA synthesis and ribosome biogenesis, nucleoid structure, transcription factories, three-dimensional superresolution Structured Illumination Microscopy, *Escherichia coli*

## Abstract

In the fast-growing *Escherichia coli* cells, RNA polymerase (RNAP) molecules are concentrated and form foci at clusters of ribosomal RNA (rRNA) operons resembling eukaryotic nucleolus. The bacterial nucleolus-like organization, spatially compartmentalized at the surface of the compact bacterial chromosome (nucleoid), serves as transcription factories for rRNA synthesis and ribosome biogenesis, which influences the organization of the nucleoid. Unlike wild type that has seven rRNA operons in the genome in a mutant that has six (Δ6*rrn*) rRNA operons deleted in the genome, there are no apparent transcription foci and the nucleoid becomes uncompacted, indicating that formation of RNAP foci requires multiple copies of rRNA operons clustered in space and is critical for nucleoid compaction. It has not been determined, however, whether a multicopy plasmid-borne rRNA operon (p*rrnB*) could substitute the multiple chromosomal rRNA operons for the organization of the bacterial nucleolus-like structure in the mutants of Δ6*rrn* and Δ7*rrn* that has all seven rRNA operons deleted in the genome. We hypothesized that extrachromosomal nucleolus-like structures are similarly organized and functional *in trans* from p*rrnB* in these mutants. In this report, using multicolor images of three-dimensional superresolution Structured Illumination Microscopy (3D-SIM), we determined the distributions of both RNAP and NusB that are a transcription factor involved in rRNA synthesis and ribosome biogenesis, p*rrnB* clustering, and nucleoid structure in these two mutants in response to environmental cues. Our results found that the extrachromosomal nucleolus-like organization tends to be spatially located at the poles of the mutant cells. In addition, formation of RNAP foci at the extrachromosomal nucleolus-like structure condenses the nucleoid, supporting the idea that active transcription at the nucleolus-like organization is a driving force in nucleoid compaction.

## Introduction

In *Escherichia coli* (*E. coli*), the growth rate is determined by growth medium (Kjeldgaard et al., 1958; Schaechter et al., 1958; Jin et al., 2012). *E. coli* cells grow rapidly in LB at 37°C with a doubling time about 20 min. In a fast-growing cell, there are multiple copies of the genome (Nielsen et al., 2007) and most RNA polymerase (RNAP) molecules engage in transcription of ribosomal RNA (rRNA) operons (French and Miller, 1989). There are seven almost identical rRNA operons which in total encompass only ∼1% of the genome, four of which are near the origin of chromosome replication *oriC.* Thus, cell size, copy numbers of bacterial chromosome (named nucleoid) and rRNA operon (*rrn*), and the organization of the nucleoid are sensitive to growth conditions (Jin et al., 2013).

Imaging RNAP-GFP using microscopy in bacterial cells has advanced our understanding of the distribution and the organization of the transcription machinery. Images of bacterial RNAP were first reported in *Bacillus subtilis* (Lewis et al., 2000), which showed that most of RNAP lies within the core of the nucleoid but is minimal at the peripheral region of the nucleoid and that, in fast-growing cells, for each nucleoid there are two RNAP foci named transcription foci at rRNA genes clusters in the *oriC* region. However, the effect of RNAP’s distribution on the organization of the nucleoid was not determined. Using advanced imaging systems and tools, extensive studies in *E. coli* have revealed that the transcription machineries not only are spatially organized but also influenced the nucleoid structure. Images of RNAP from these two bacteria share similarities but also reveal differences. These differences could be due to microbial diversity, and/or different cell imaging techniques used in different studies. In *E. coli* cells grown in nutrient rich LB, RNAP molecules are concentrated and form foci at clusters of rRNA operons resembling eukaryotic nucleolus-like structure (Cabrera and Jin, 2003). The 3D images of multicolor superresolution Structured Illumination Microscopy (3D-SIM) reveal that under optimal growth conditions (LB at 37°C), RNAP foci, spatially located at the periphery of the compact nucleoid, co-localize with transcriptional factors, NusA and NusB, both of which are involved in rRNA synthesis and ribosome biogenesis (Greenblatt and Li, 1981; Torres et al., 2004; Greive et al., 2005; Stagno et al., 2011; Bubunenko et al., 2013). RNAP foci thus represent transcription factories (Cook, 2010; Papantonis and Cook, 2013) for the expression of growth genes, analogous to the eukaryotic Pol I activity at the nucleolus in the nucleus (Jin et al., 2017). Such an organization of hyperstructure (Norris et al., 2007) would considerably facilitate RNAP recycling and recruitment for synchronized active rRNA synthesis, rRNA processing and ribosome assembly in spatial proximity. However, RNAP is mobile, and RNAP foci and the associated macromolecular organization are dynamic and sensitive to environmental cues (Bakshi et al., 2012; Endesfelder et al., 2013; Jin et al., 2013, 2015; Stracy et al., 2015). For example, the transcription foci and possibly the nucleolus-like structure disappear, leading to an expanded nucleoid when growth is slowing down or arrested during stress responses (Cabrera and Jin, 2003), such as by amino acid starvation, which induces the stringent response (Cashel et al., 1996; Durfee et al., 2008) or with rifampicin treatment, which inhibits transcription initiation. A strong, positive correlation between the presence of transcription foci and the occurrence of relatively compact states of the nucleoid demonstrates an interconnection of the organizations of transcription machinery and the nucleoid (Jin et al., 2013). However, determining whether transcription associated with the bacterial nucleolus-like structure or organization (hereafter they are used interchangeably) plays a direct role in the compaction of the nucleoid has been difficult to dissect.

Having multiple copies of rRNA operons in the bacterial genome is a prerequisite for the formation and organization of RNAP foci in *E. coli*. A series of chromosomal *rrn* deletion(s) has been constructed and characterized (Condon et al., 1993, 1995; Quan et al., 2015). *E. coli* strains in which two out of seven rRNA operons were deleted in the genome behave like wild type in growth rate, formation and organization of RNAP foci, and nucleoid structure. However, mutant strains in which additional multiple chromosomal rRNA were deleted reduce growth rate and cause changes in the distribution of RNAP and nucleoid structure (Jin et al., 2016). For example, in the mutant strain (Δ6*rrn*), in which six out seven rRNA operons were deleted from the genome, there are no apparent transcription foci and the nucleoid becomes uncompacted under optimal growth conditions (LB at 37°C). These phenotypes of Δ6*rrn* can be explained by reduced rRNA synthesis and ribosome biogenesis due to the lack of bacterial nucleolus-like organization in the cell (Cabrera et al., 2009). Thus, this genetic background has provided a unique system to address the potential role of bacterial nucleolus-like organization supplemented *in trans* by a multicopy plasmid-borne rRNA operon in nucleoid compaction. Previously it was briefly reported (Jin et al., 2017) that the RNAP foci reappear in the mutant strain that harbors pKK3535 (in which the *rrnB* operon is cloned into pBR322 vector; hereafter, this plasmid-borne rRNA operon is referred to as p*rrnB*) (Kingston et al., 1981). However, the organization of the transcription machinery and the distribution of p*rrnB* have not been determined. In this study, we characterized the structure and function of RNAP foci in the mutants of Δ6*rrn*/p*rrnB* and Δ7*rrn*/p*rrnB*, in which all seven chromosomal rRNA operons were deleted. Our results showed that analogous to wild type, RNAP forms foci at clusters of p*rrnB* for rRNA synthesis and ribosome biogenesis in the two mutants, and suggested that active transcription at the extrachromosomal nucleolus-like organization is a driving force in nucleoid compaction.

## Materials and Methods

### Bacterial Strains, Growth Conditions, and Technologies

All of the strains used in the study are derivatives of the prototype K-12 MG1655. **Table 1** lists the strains and plasmids used in the study. The MG1655 strain has a doubling time 20 min in LB at 37°C (Jin et al., 2013), as measured by determining the increase of OD at 600 nm (OD_600_) of bacteria cultures in a shaking flask as a function of time. The chromosomal *rpoC-venus* and *nusB-mCherry* fusions were described previously (Cagliero and Jin, 2013; Cagliero et al., 2014) and have no effect on cell growth. They were introduced to different strains either by P1 phage transduction or by phage lambda Red recombineering (Datsenko and Wanner, 2000). The bacterial media and techniques used are described elsewhere (Miller, 1972). All cultures were grown in Luria-Bertani medium (LB which is tryptone 10 g/l, yeast extract 5 g/l, NaCl 5 g/l) with vigorous agitation in a water bath at 37°C. Overnight cultures were diluted 1/200 into fresh medium. Samples used for microscopic observation were removed from cultures at an OD 0.2 at 600 nm (OD_600_), and when indicated, rifampicin (150 μg/ml), chloramphenicol (200 μg/ml), and freshly made serine hydroxamate (SHX; 100 mM) were added to the log phase cultures at time zero, and sampled at indicated times. These inhibitors stopped cell growth almost immediately. Media downshifts (from LB to minimal medium) were performed by filtering the exponentially growing culture after the absorbance reached OD_600_ 0.2. Cells were then collected on a nitrocellulose Millipore HA WP04700 filter (47 mm diameter with a 0.45 μm pore diameter). The filtration was performed using a vacuum pump (Millipore), allowing a minimal amount of time to obtain the new condition (about 1 min). Filtered cells were washed with 5X filtered-volume of the minimum medium, followed by resuspension (vortexing) for 1 min in the same volume of pre-heated growth minimum medium (as time zero). Later, incubation was performed under the same shaking and temperature conditions. All materials used were preheated at incubation temperature (at 37°C) to avoid abrupt changes in temperature. The downshift minimal medium is a supplement of M63x salt, 0.01 M CaCl_2_, 0.1 M MgSO_4_, and 0.4% glucose. The antibiotics and chemicals were from Sigma.

**Table 1:** Bacterial strains and plasmids.

Strains	Relevant Genotype	Source/Reference
CC72	MG1655 *rpoC-Venus*	Cagliero and Jin, 2013
SQZ5	MG1655 Δ6*rrn* [(Δ*rrn*GADEHB)(ptRNA67)]	Cabrera et al., 2009
CC125	SQZ5 *rpoC*-Venus	This work
CC126	CC125(pKK3535)	This work
CC378	CC125(pBR322)	This work
SQ171	MG1655 Δ7*rrn* [(Δ*rrn*GADEHBC)(ptRNA67, pKK3535)]	Quan et al., 2015
CC379	SQ171 *rpoC*-Venus	This work
CC382	CC125 *nusB-mCherry::Kan*	This work
CC383	CC126 *nusB-mCherry::Kan*	This work
CC384	CC378 *nusB-mCherry::Kan*	This work
CC385	CC379 *nusB-mCherry::Kan*	This work
CC386	CC125(pTac-16S)	This work
CC387	CC125[pMKA201(pT7-rpoC)]	This work
CC389	CC125(pK4-16)	This work

**Plasmids**	**Description**	**Source/Reference**

ptRNA67	tRNA genes cloned in p15 replicon	Cabrera et al., 2009
pKK3535	*rrnB* operon cloned in pBR322	Kingston et al., 1981
pBR322	cloning vector	Bolivar et al., 1977
pDJ2485	pTac-16S (16S rRNA gene inserted into pKK223-3)	Cabrera and Jin, 2006
pMKA201	pT7-rpoC in pTZ19R	Kashlev et al., 1993
pK4-16	rrnB operon cloned in pSC101	Quan et al., 2015


### 3D Superresolution Microscopy

Because both the distribution of *E. coli* RNAP and the nucleoid structure are extremely sensitive to perturbations in the environment, cells were immediately fixed using formaldehyde (3.7% v/v final) after sampling (Cabrera and Jin, 2003). It is critical and mandatory to use fixed cells on slides to study the dynamic organization of RNAP and DNA in fast growing cells under optimal growth conditions (Jin et al., 2015). The procedure of culture sampling and multicolor 3D SIM imaging was as described (Mata Martin et al., 2018). The 3D superresolution microscopy was performed with a Nikon N-SIM Ti-2E inverted microscope with a CFI SR HP Apochromat TIRF 100XC Oil (NA 1.49) objective and LU-NV series laser units. Images with a high signal-to-noise ratio were captured with a high-resolution ORCA-Flash 4.0 sCMOS camera (Hamamatsu Photonics K.K.). We performed three grid rotations per image and at least 15 *z*-sections of 0.1 μm to acquire the whole cells. The exposure time and setting are determined by the fluorescence proteins. After image acquisition, images were processed to correct for chromatic aberration using the Software NIS-Elements Ar/NIS-Elements C using the inframe calibration beads for optimum alignment, and were reconstituted.

The cells edited and illustrated are representative of the majority of the observed cells. Pictures were processed uniformly using FijiJ to crop and choose the best *z*-slice or maximum intensity projection slide and were false-colored with Adobe Photoshop.

### Image Analyses

Relative nucleoid size (RNS) [ratio of size of nucleoid(s) over size of the cell] was measured as described (Cabrera et al., 2009). For each condition, 100 cells were analyzed. Contrast analysis of the distribution of RNAP in nucleoids was described (Cabrera and Jin, 2003). A Java applet named Nucleoid Analyzer was written for the measurements. At each position in the nucleoid, the Venus fluorescence signal is proportional to the concentration of RNAP. The homogeneity in the RNAP distribution can be evaluated by measuring the differences in fluorescence signals between each position of the nucleoid and its neighboring positions. In nucleoids in which RNAP is not distributed homogeneously (nucleoids with transcription foci), there will be more differences on average in the fluorescence signal between neighboring positions than in nucleoids in which the RNAP is distributed homogeneously (nucleoids without transcription foci). For each growth condition, nucleoids of > 100 cells were analyzed and the data were presented as normalized contrast, as described (Cabrera and Jin, 2003). Contrast analysis of the distribution of DNA signals was similarly measured and presented. The measurement of distribution profiles in a population of cells was performed in FijiJ by Analyzer Plot Profile. Cells were segmented, giving a cell outline and a cell midline. The distances were normalized to 1 and -1 at the cell midline from the outline in one pole to the outline in the opposite pole. The 2D histograms of the distributions were generated by binning cells into different lengths (3.1–4.6 m long); they indicate the probability density distribution of a body in the cell across the *X*-axis for 100 cells.

### Live-Cell Imaging Using a Microfluidics System

Overnight cells from 32°C in LB (16 h) were injected into an ONIX Microfluidic Perfusion System (CellASIC) and were allowed to grow at 32°C in LB with a pressure of 2 psi. The cells were growing in the microfluidic device with a continuous flow of fresh LB; the temperature was controlled by Zeiss control temperature module S. The time-lapse images were acquired using an inverted microscope (Zeiss Axio Observer) with a Plan Apochromat 100 × /1.4 oil phase objective and a 1.6 Optovar, with a high signal-to-noise ratio and high-resolution EMCCD camera (Hamamatsu). Each experiment was performed at least three times; in total, 37 cells for each condition were imaged. The cells illustrated are representative observed cells. Pictures were processed uniformly and were false-colored with Adobe Photoshop.

### Cell Lysis and Imaging Released Nucleoids and Transcription Factories

Immediately after sampling, the cells were fixed for 20 min at room temperature with formaldehyde (3.7% v/v final). The cells were centrifuged at 5000 *g* for 5 min; resuspended in 50 μl of 10 mM Tris (pH 8.0), 10 mM EDTA, 20% sucrose, 0.2 μl Ready-lyse lysozyme (Epicenter); and incubated for 30 min at 37°C. The cell lysis was completed by the addition of 50 μl of water. After lysis, a 2-μl aliquot of the lysate containing nucleoids was dropped onto a microscopy slide with 2 μl of 1% warm low melting point agarose + 10 μg/ml Hoechst 33342. The slides were imaged immediately with YFP channel for RNAP-Venus, DAPI channel for DNA, mCherry channel for NusB-mCherrry using an inverted microscope (Zeiss Axio Observer) with a Plan Apochromat 100 × /1.4 oil phase objective and a 1.6 Optovar, with a high signal-to-noise ratio and high-resolution EMCCD camera (Hamamatsu).

### RNA-Fluorescence *in Situ* Hybridization (FISH)

The RNAI was used as a probe to observe the subcellular localization of the plasmid-borne p*rrnB*. The different probes cover all RNAI regions. Probe design was based on the protocol described by Raj et al. (2008) and Raj and Tyagi (2010). Each probe was ordered with a 3′ amine group, which allows covalent modification with NHS-ester derivatives of fluorescent dye molecules and were labeled with red fluorescence compound Atto 594 NHS ester as described previously (Skinner et al., 2013). Fluorescence *in situ* hybridization was carried out according to the protocol reported previously (Skinner et al., 2013). Briefly, cells were fixed using formaldehyde (37%) and PBS (10%) for 30 min. Fixed cells were centrifuged at 4000 rpm for 5 min and washed three times in PBS and subsequently incubated in 70% Ethanol for 60 min at room temperature. Cells were washed three times in a washing solution (2xSSC and 40% formamide in DEPC water) for 5 min at 37°C. After washing the cells were incubated with the probes in hybridization buffer (2xSSC, 40% formamide, 10% dextran sulfate in DEPC water) overnight at room temperature. After incubation overnight, the cells were washed three times in the washing solution and three times in 2xSSC. A cell suspension was applied to the slides for imaging. All images were imaged and processed as described previously in the Section “3D Superresolution Microscopy.”

### Plasmid Copy Number Determination by qRT-PCR

The method was used as previously described (Lee et al., 2006; Anindyajati et al., 2016). Briefly, the single-copy gene *tdk* and *bla* on the chromosome and pKK3535, respectively, were chosen to quantify the absolute plasmid copy number. The *tdk* gene was amplified by PCR and cloned into a pBAD24 plasmid to produce pBAD24-*tdk*, which also has one copy of *bla* gene in the backbone. A 10-fold serial dilution series of the plasmid pBAD24-*tdk* extracted from *E. coli*, ranging from 1 × 10^-4^ to 1 × 10^-9^ copies/μl, were used to make the standard curve for the *tdk* and *bla* genes. Then the pKK3535 plasmid and genome DNA were extracted from Δ6*rrn* and Δ6*rrn*/p*rrnB* grown in LB at 37°C by Wizard^®^ Genomic DNA Purification Kit (Promega), and diluted to 2 ng/μl. Quantification of the chromosome (*tdk*) and plasmid (*bla*) were performed using SYBR^®^ Green based qRT-PCR. Using the *C*t values, the absolute quantities of the chromosome and plasmid were determined according to the standard curve. The plasmid copy number was then calculated by dividing the above quantity of the plasmid by the amount of the chromosome.

## Results

### Extrachromosomal Nucleolus-Like Compartmentalization by a Multicopy Plasmid-Borne Ribosomal RNA Operon

#### RNAP Foci Organization Independent of Chromosomal rRNA Operons

**FIGURE 1:**
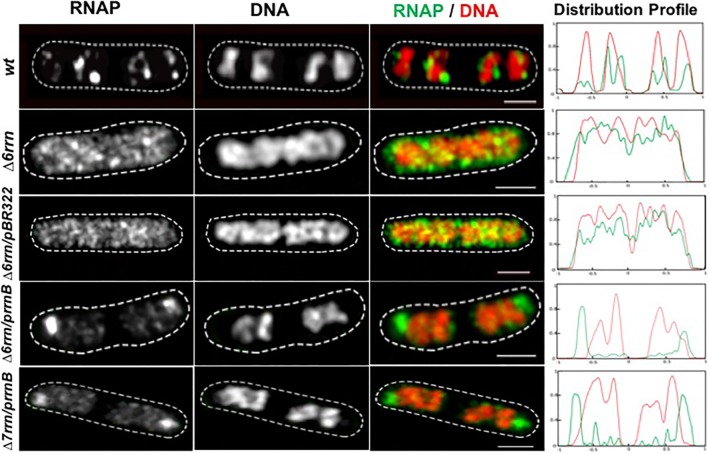
Compartmentalization of RNAP foci and nucleoid condensation caused by the plasmid-borne p*rrnB* in Δ6*rrn* and Δ7*rrn* mutants. Shown are SIM images of RNAP, DNA, and an overlay RNAP (green) and DNA (red) in representative single cells during optimal growth (LB, 37°C). Wild type (*wt*) is included as a control. RNAP is tagged with yellow Venus fluorescent protein (*rpoC*-venus) and DNA is stained with Hoechst 33342, as described in the Section “Materials and Methods.” Note that the defects in the Δ6*rrn* or Δ7*rrn* strains in the organization of RNAP foci and nucleoid structure can be complemented by a plasmid-borne rRNA operon (p*rrnB*) in those mutant cells. RNAP foci mainly locate at the cell poles in the two mutants. The cell shape is outlined by a dotted line. The scale bar represents 1 μm. The two-color graphics show the distribution profiles of RNAP and nucleoid in a population of 100 cells; *Y*-axis is the probability density of RNAP (green line) and DNA (red line) across the cell length (*X*-axis) from one pole (–1) to another pole (+1).

Previously, a comparison was made only between the Δ6*rrn* and Δ6*rrn*/p*rrnB* strains (Jin et al., 2017). Because in pKK3535 (Kingston et al., 1981) the *rrnB* operon was cloned into the pBR322 vector and to eliminate a potential effect by the vector, we introduced the pBR322 into Δ6*rrn* (Δ6*rrn*/pBR322), and examined the phenotypes of the two strains, Δ6*rrn*/pBR322 and Δ6*rrn*/p*rrnB*, under optimal growth conditions (LB at 37°C). We found no differences between Δ6*rrn* and Δ6*rrn*/pBR322, both of which have the same generation time (τ= 37 min). The generation time for Δ6*rrn*/p*rrnB* is longer (τ= 43 min), indicating that p*rrnB* did not suppress the growth defect of the Δ6*rrn* mutant. The slower growth rate of Δ6*rrn*/p*rrnB* compared to Δ6*rrn*/pBR322 is likely due to an unbalanced growth condition caused by the p*rrnB*. SIM images show that there are no RNAP foci in either Δ6*rrn* or Δ6*rrn*/pBR322 strains and the nucleoid is expanded in those cells; however, RNAP foci are evident in Δ6*rrn*/p*rrnB* (**Figure 1**), indicating that the formation of RNAP foci is caused by the plasmid-borne *rrnB*. Unlike wild type, in which multiple RNAP foci are spatially located at the surface of four nascent nucleoids, in Δ6*rrn*/p*rrnB*, there are usually two larger RNAP foci, which are primarily located at the cell poles. To eliminate a potential effect of the remaining chromosomal *rrnC* in the Δ6*rrn* mutant cells on the formation of RNAP foci, we examined the Δ7*rrn*/p*rrnB* mutant cells, in which all seven rRNA operons were deleted in the genome. Like Δ6*rrn*/p*rrnB*, usually two large RNAP foci are apparent and located at cell poles in Δ7*rrn*/p*rrnB* (**Figure 1**), indicating that the formation/organization of RNAP foci is independent of chromosomal rRNA operons but is associated with the plasmid-borne *rrnB*. Each of the Δ6*rrn*/p*rrnB* and Δ7*rrn*/p*rrnB* cells on average has two apparent nascent nucleoids which are compact.

**FIGURE 2:**
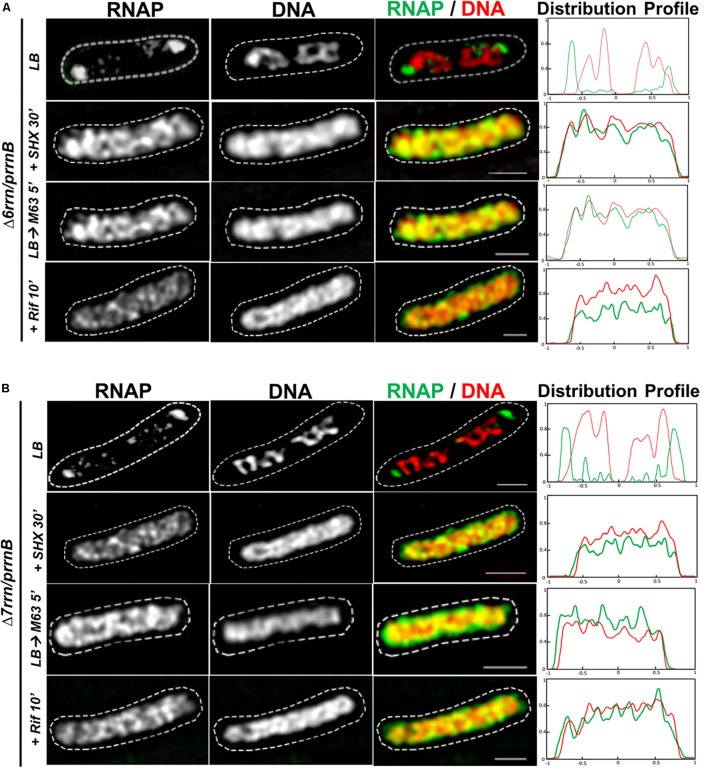
Organizations of RNAP foci and the nucleoids are sensitive to changes in growth medium and/or stress. Shown are SIM images of RNAP, DNA, and two-color overlays of RNAP (green) and DNA (red), from representative single cells of Δ6*rrn/*p*rrnB* **(A)** and Δ7*rrn/*p*rrnB* **(B)** during optimal growth (LB) and after perturbations induced by SHX treatment for 30 min (+SHX 30′), nutrient down shift from LB to minimal medium M63+glucose for 5 min (LB→M63 5′), and after rifampicin treatment for 10 min (+Rif 10′). The cell shape is outlined by a dotted line. The scale bar represents 1 μm. The two-color graphics show the distribution profiles of RNAP and nucleoid in a population of 100 cells, as described in the **Figure 1** caption.

Like their counterparts in wild type, formation/organization of RNAP foci in the Δ6*rrn*/p*rrnB* and Δ7*rrn*/p*rrnB* mutants is also sensitive to environmental cues (**Figures 2A,B**). For example, RNAP foci disappear when the mutant cells were downshifted from LB to minimal medium or starved for amino acids by the addition of SHX. The synthesis of rRNA in cells is significantly reduced by these two treatments. Similarly, there were no RNAP foci when cells were treated with antibiotic rifampicin that inhibits transcription (re)initiation. In addition, similar to wild type, the nucleoids become expanded in those stressed cells compared to those in cells under optimal growth conditions (see details below).

#### Colocalization of RNAP Foci and Clusters of p*rrnB*

**FIGURE 3:**
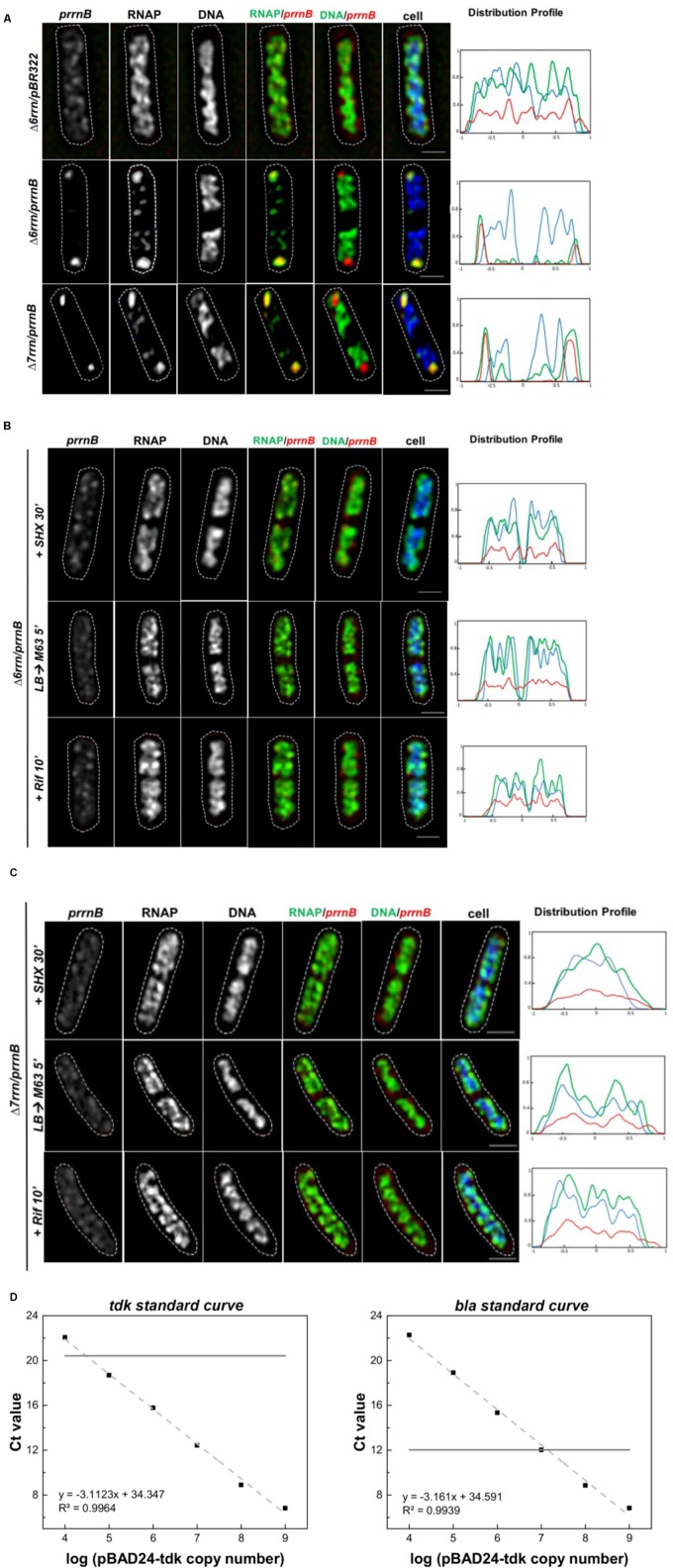
Active transcription of p*rrnB* promotes coordinated organization of RNAP foci and p*rrnB* clusters. **(A–C)** RNA-Fluorescence *in situ* hybridization (FISH). Shown are SIM images of p*rrnB*, RNAP, DNA, two-color overlays of RNAP (green) and p*rrnB* (red), DNA (green) and p*rrnB* (red), and three-color overlays of p*rrnB* (red), RNAP (green) and DNA (blue) from representative single cells during optimal growth (LB) **(A)**, and **(B,C)** after perturbations induced by SHX treatment for 30 min (+SHX 30′), nutrient down shift from LB to minimal medium M63+glucose for 5 min (LB→M63 5′) and after rifampicin treatment for 10 min (+Rif 10′). The distribution of p*rrnB* is determined by RNA-FISH as described in the Section “Materials and Methods.” The cell shape is outlined by a dotted line. The scale bar represents 1 μm. The three-color graphics show the distribution profiles of RNAP, nucleoid and p*rrnB* in a population of 100 cells; *Y*-axis is the probability density of the plasmid p*rrnB* (red line), RNAP (green line), nucleoid DNA (blue line) across the cell length (*X*-axis) from one pole (–1) to another pole (+1). Note that p*rrnB* clusters and RNAP foci perfectly colocalize (overall yellow color on the RNAP/p*rrnB* overlay) and are located near the cell poles during optimal growth **(A)**; however, the distributions of both p*rrnB* and RNAP become random without foci and clusters in stressed cells **(B,C)**. **(D)** Plasmid copy number determination by qRT-PCR. A 10-fold serial dilution series of the plasmids pBAD24-*tdk* were used to make the standard curves for the *tdk* and *bla* genes, respectively (dash lines). The pKK3535 plasmid and genome DNA extracted from the strain (Δ6*rrn* and Δ6*rrn*/p*rrnB*) were used by qRT-PCT to determine the *C*t values of *tdk* from genome and *bla* from pKK3535 (solid lines). Based on the differences of the two *C*t values, the copy numbers of pKK3535 relative to genome were calculated, as described in the Section “Materials and Methods.”

To determine whether RNAP foci are associated with the plasmid-borne p*rrnB*, we attempted to co-image RNAP and the plasmid in the Δ6*rrn*/p*rrnB* and Δ7*rrn*/p*rrnB* cells. Our first approach using DNA-FISH assay failed because of the harsh conditions required in the protocol, including high temperatures, destroyed RNAP foci and the nucleoid structure. We then successfully used the RNA-FISH method, which used mild conditions, to detect the location of p*rrnB* by hybridization of fluorescent DNA probes of the RNAI transcripts (Morita and Oka, 1979) made from PBR322 vector portion. We chose RNAI as a tag for the location of p*rrnB* because RNAI targets the replication region of the plasmid (Davison, 1984). Our results showed that, like RNAP’s distribution, the distribution of the plasmid-borne p*rrnB* is sensitive to growth conditions. Under optimal growth conditions, there are primarily two clusters of p*rrnB*, which are located at the cell poles and are colocalized with RNAP foci in the mutants (**Figure 3A**). In contrast, signals of pBR322 from the RNA-FISH are scattered in the Δ6*rrn*/pBR322 cells. In addition, both RNAP foci and p*rrnB* clusters are absent in the cells of stressed mutants, caused by nutrient downshift, amino acid starvation, and rifampicin treatment (**Figures 3B,C**). We concluded from these results that active rRNA synthesis promotes the clustering of plasmid-borne p*rrnB* in the mutants.

We also determined the relative copy number of the plasmid p*rrnB* compared to a single-copy chromosomal gene (*tdk*) in the Δ6*rrn*/p*rrnB* cells grown in LB at 37°C, and found that, on average, there are 12.0 ± 1.2 copies of p*rrnB* per genome (**Figure 3D**), or about 24 copies of p*rrnB* per cell because most of the cells have two nascent nucleoids per cell as revealed by cell images (**Figure 1**). Thus, it is estimated that each of the two RNAP foci is organized at a p*rrnB* cluster of about 12 copies. This could explain why the RNAP foci appear to be larger in the two mutants than those RNAP foci in wild type, in which each of RNAP foci locates at a cluster of about six rRNA operons (Cagliero et al., 2014). We concluded that in both Δ6*rrn*/p*rrnB* and Δ7*rrn*/p*rrnB* mutants, RNAP foci are located at clusters of plasmid-borne *rrnB* resembling extrachromosomal bacteria nucleolus-like organization.

#### Colocalization of the Foci of RNAP Foci and NusB That Participate in rRNA Synthesis and Ribosome Biogenesis

**FIGURE 4:**
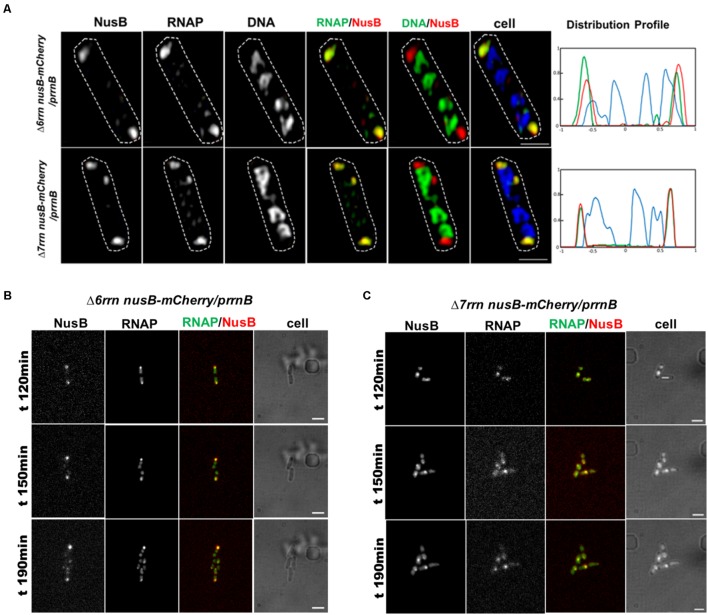
Foci of nascent rRNA-binding protein NusB colocalizes with RNAP foci in the Δ6*rrn/*p*rrnB and Δ*7*rrn/*p*rrnB* mutants containing the chromosomal *nusB-mCherry* fusion **(A)**. Shown are SIM images of NusB, RNAP, DNA, two-color overlays of RNAP (green) and NusB (red), DNA (green) and NusB (red), and three-color overlays of RNAP (green), NusB (red) and DNA (blue) from representative single cells under optimal growth conditions (LB, 37°C). NusB is tagged with m-Cherry fluorescent protein in the two mutant chromosomes. Note that NusB foci are at the cells poles, and the NusB foci signals perfectly colocalize with RNAP foci signals (overall yellow color on the RNAP/NusB overlay). The cell shape is outlined by a dotted line. The scale bar represents 1 μm. The three-color graphics show the distribution profiles of NusB, RNAP and nucleoid in a population of 100 cells; *Y*-axis is the probability density of NusB (red line), RNAPs (green line) and nucleoid (blue line) across the cell length (*X*-axis), as described in the **Figure 1** caption. Live-cells imaging of Δ6*rrn nusB-mCherry/*p*rrnB* **(B)** and Δ7*rrn nusB-mCherry/*p*rrnB* **(C)** using microfluidics confirms that RNAP foci and NusB colocalize at the cell poles in the mutants containing the chromosomal *nusB-mCherry* fusion. Cells were growing in LB using the CellASIC ONIX microfluidic system, as described in the Section “Materials and Methods.” Note that RNAP foci and NusB foci are colocalized at the cell poles, and the changes in the cells position and size during the imaging process. The scale bar represents 2 μm.

In wild type, RNAP foci at the bacterial nucleolus-like structure represent transcription factories for rRNA synthesis and ribosome biogenesis because they co-localize with the foci of NusA and NusB (Cagliero et al., 2014; Jin et al., 2017), two transcription factors involved in those processes (Greenblatt and Li, 1981; Torres et al., 2004; Greive et al., 2005; Stagno et al., 2011; Bubunenko et al., 2013). To determine whether RNAP foci also associated with the Nus factors in both Δ6*rrn*/p*rrnB* and Δ7*rrn*/p*rrnB* mutants, we chose to co-image RNAP with NusB that binds to nascent rRNA for rRNA processing. SIM images revealed that NusB foci are apparent and they are colocalized with RNAP foci at the cell poles in the two mutants (**Figure 4A**). Live-cell imaging using a microfluidic system also confirmed colocalization of the foci of RNAP and NusB at the cell poles (**Figures 4B,C**). These results indicate that, despite differences in spatial locations, the compositions of the extrachromosomal bacteria nucleolus-like organization in the two mutants are likely to be the same as that of the bacterial nucleolus-like structure in wild type (Jin et al., 2017). Because p*rrnB* supports cell growth in the Δ7*rrn*/p*rrnB* mutant, we concluded that transcription factories at the extrachromosomal bacteria nucleolus-like organization are fully functional for rRNA synthesis and ribosome biogenesis.

### Long-Range Interaction Between RNAP Foci at Extrachromosomal Nucleolus-Like Organization and the Bacterial Chromosome

#### Condensed Nucleoids in the Mutants Which Have RNAP Foci Associated With p*rrnB* Clusters

**FIGURE 5:**
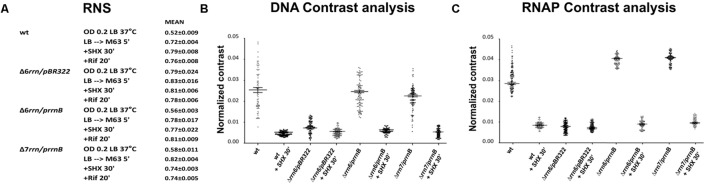
Image analyses of the relative nucleoid size, nucleoid organization, and the distribution of RNAP in different strains under different growth conditions. **(A)** Mean values of relative nucleoid size (RNS) of indicated strains under optimal growth (LB, 37°C) and indicated stressed conditions from a population of 100 cells. RNS was measured as described in the Section “Materials and Methods.” Larger values of RNS indicate more expended nucleoids in the cells. **(B)** Normalized contrast parameters of the distributions of DNA signals in indicated strains under different growth conditions. The normalized contrast parameters were measured from 100 nucleoids as described in the Section “Materials and Methods.” In this analysis, larger values indicate more heterogeneous distribution of DNA signals with structured features, and are correlated with compact nucleoids. In contrast, smaller values of normalized contrast indicate more homogenous distribution with less structured features, and are correlated with larger values of RNS. **(C)** Normalized contrast parameters of the distributions of RNAP signals in indicated strains under different growth conditions. The normalized contrast parameters were measured from 100 cells as described in the Section “Materials and Methods.” In this analysis, larger values of normalized contrast indicate more heterogeneous distributions of the RNAP, which in turn are indicative of the presence of transcription foci. In contrast, smaller values indicate that RNAP is more homogeneously distributed and that there is a lack of RNAP foci.

We next addressed questions regarding the effect of RNAP foci at the extrachromosomal nucleolus-like organization on nucleoid organization *in trans* in the Δ6*rrn*/p*rrnB* and Δ7*rrn*/p*rrnB* mutants. In wild type, transcription and the distribution of RNAP link to the organization of the nucleoid (Cabrera and Jin, 2003; Jin and Cabrera, 2006; Jin et al., 2013). We used several approaches to determine whether RNAP foci at the extrachromosomal nucleolus-like structure also influence the organization of the nucleoid in these two mutants. First, we measured relative nucleoid size (RNS) [ratio of size of nucleoid(s) over size of the cell], as previously described (Cabrera et al., 2009). In the control Δ6*rrn*/pBR322 cells in which there is no RNAP foci (**Figure 1**), the nucleoids are expanded (**Figure 5A**) with a mean RNS value of 0.79; the distributions of DNA and RNAP are relatively homogeneous, as indicated by contrast analyses (Cabrera and Jin, 2003) (**Figures 5B,C**). In contrast, in the Δ6*rrn*/p*rrnB* and Δ7*rrn*/p*rrnB* cells, RNAP foci are apparent (**Figure 1**) and the nucleoids are compact with a small RNS value of about 0.56–0.58, and the distributions of DNA and particularly RNAP are highly heterogeneous (**Figures 5B,C**) due to the presence of RNAP foci at the cell poles.

Second, we determined the effects of stresses on the formation of RNAP foci, the organization of extrachromosomal nucleolus-like structure and the nucleoid organization. As described above, cells were stressed when the nutrient downshifted, were starved for amino acid, or were treated with rifampicin. Under those stress conditions, RNAP foci are absent (**Figures 2A,B**) and the nucleoids become expanded with increased values of RNS (**Figure 5A**), and the distributions of DNA and RNAP converted to relatively homogeneous states compared to cells prior to the stresses (**Figures 5B,C**). Because the disappearance of RNAP foci is also associated with the dispersing of the clusters of p*rrnB* in the stressed mutants as described above (**Figures 3B,C**), we concluded that active transcription at the extrachromosomal nucleolus-like structure is required for the nucleoid compaction.

#### Compacted Nucleoids and Transcription Factories Released From Lysed Mutant Cells

**FIGURE 6:**
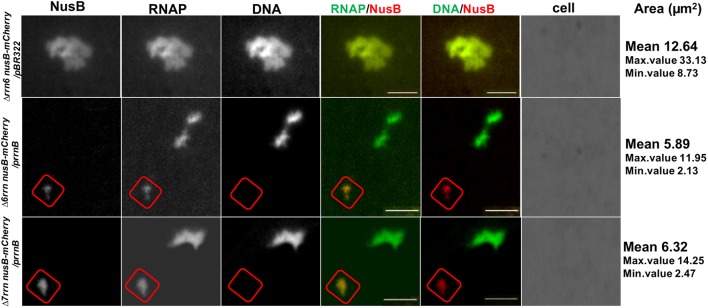
Released nucleoids and transcription factories for rRNA synthesis and ribosome biogenesis from lysed mutant cells. Cells of indicated strains grown in LB at 37°C were lysed followed by coimaging of NusB, RNAP, DNA in released nucleoids, and transcription factories and cells using wide field epifluorescence microscopy, as described in the Section “Materials and Methods.” Shown are images of NusB, RNAP, and DNA, and overlays of RNAP (green) and NusB (red) and DNA (green) and NusB (red). No intact cells were visualized because they were completely lysed. Note that there are very low NusB signals in the nucleoid of the Δ6*rrn nusB-mCherry/*p*rrnB* and Δ7*rrn nusB-mCherry/*p*rrnB* cells, in contrast to that in Δ6*rrn nusB-mCherry/*pBR322. The cycles indicated putative transcription factories, which have strong colocalized signals of NusB and RNAP but very low DNA signals, and they are present only in cells harboring the p*rrnB*. The scale bar represents 2 μm. Area mean (μm^2^), maximum and minimum values of the areas of 100 isolated nucleoids from different strains are indicated in the last column of the figure. Smaller areas indicate more compact organizations of the nucleoids.

We also examined isolated nucleoids by co-imaging RNAP and DNA from lysed cells of different strains (**Figure 6**). The nucleoids released from the Δ6*rrn nusB-mCherry* /pBR322 cells are expanded with large areas (2D images), and are associated with RNAP and NusB. In contrast, the nucleoids released from the Δ6*rrn nusB-mCherry /*p*rrnB* and Δ7*rrn nusB-mCherry /*p*rrnB* cells lysates are compact with much smaller areas, and are associated with RNAP, but not with NusB, suggesting weak interactions between DNA and NusB. In addition, released RNAP foci from those cells lysates are evident as indicated by having strong RNAP-Venus signals but minimal DNA signals, indicating that RNAP foci associated with the extrachromosomal nucleolus-like structure can be physically separated from the nucleoid. The isolated RNAP foci are also colocalized with the foci of NusB, demonstrating that they are isolated transcription factories for rRNA synthesis and ribosome biogenesis.

#### Hypercondensed Nucleoid in the Mutant Cells Treated With Chloramphenicol

**FIGURE 7:**
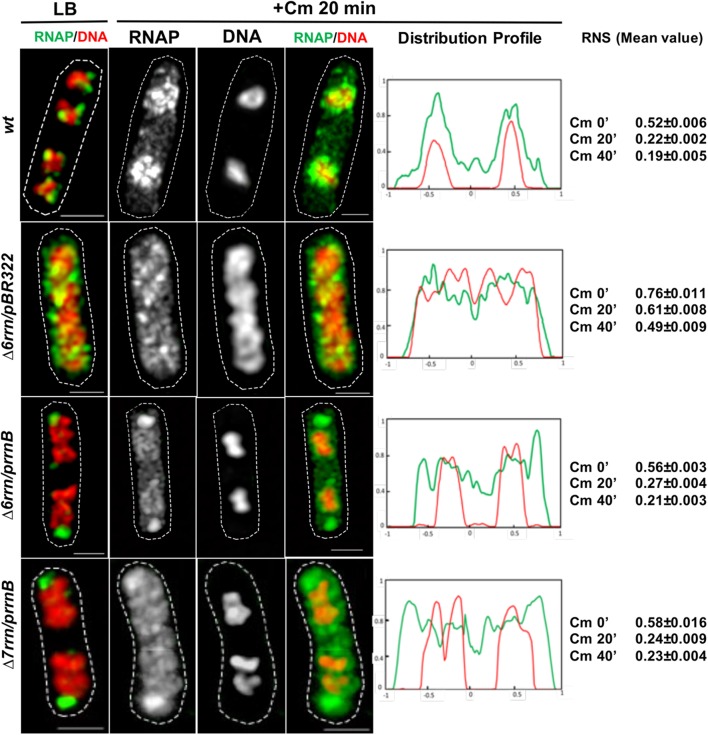
Active transcription of the extrachromosomal nucleolus promotes hyper-condensation of the nucleoids in cells treated with chloramphenicol mimicking wild type (*wt*). Shown are SIM images of indicated cells before and after chloramphenicol treatment. For simplicity, only an overlay RNAP (green) and DNA (red) is shown prior to the antibiotic treatment (LB) because **Figure 1** includes more detailed sets of images. Images of RNAP and DNA and an overlay RNAP (green) and DNA (red) are shown in cells treated with the antibiotic for 20 min. As a control, *wt* is included in the experiments. The cell shape is outlined by a dotted line. The scale bar represents 1 μm. The two-color graphics show the distribution profiles of RNAP (green) and DNA (red) in a population of 100 cells 20 min after the antibiotic treatment, as described in the **Figure 1** caption. Mean values of relative nucleoid size (RNS) in different cells population (100 cells) before, and at 20 and 40 min after chloramphenicol treatment are also shown. Images and image analyses show that, as in *wt*, the nucleoids become hyper-condensed in the Δ6*rrn/*p*rrnB* and Δ7*rrn/*p*rrnB* cells, in contrast, the nucleoids are still relatively expanded in Δ6*rrn/pBR322* after the chloramphenicol treatment.

To provide further evidence that transcription activity of the extrachromosomal nucleolus-like organization drives nucleoid compaction, we utilized another phenotype of the two mutants after chloramphenicol treatment, which is known to condense nucleoids (van Helvoort et al., 1996; Zimmerman, 2002). It was reported that due to reduced rRNA synthesis, the nucleoid is less condensed after chloramphenicol treatment in the Δ6*rrn* mutant cells compared with wild type (Cabrera et al., 2009). We repeated the experiments with the Δ6*rrn*/pBR322 strain and obtained similar results. We then examined the effect of RNAP foci on the nucleoid compaction in Δ6*rrn*/p*rrnB* and Δ7*rrn*/p*rrnB* after chloramphenicol treatment (**Figure 7**). Our results show that the nucleoids become hyper-condensed after chloramphenicol treatment in the two mutants. This is the same result as wild type but different from Δ6*rrn*/pBR322. For example, image analyses showed that 20 min after the addition of the antibiotic, the RNS value has decreased more than twofold from 0.56 (prior to the treatment) to 0.27 in Δ6*rrn*/p*rrnB* cells. In contrast, in the control Δ6*rrn*/pBR322 cells, the RNS value has reduced only about 1.25-fold from 0.76 (prior to the treatment) to 0.61, 20 min after the treatment, and reduced only about 1.55-fold (0.76 vs. 0.49) 40 min after the addition of chloramphenicol. Similar results were obtained in the Δ7*rrn*/p*rrnB* cells. We concluded that RNAP foci associated with the extrachromosomal nucleolus-like structure *in trans* has a long-range effect in compacting the nucleoid.

There is an intriguing observation indicating that the RNAP–DNA interaction is altered in cells treated with chloramphenicol. We found that, similar to wild type, in both mutants treated with chloramphenicol there are apparent “free” or dissociated RNAP in the cytoplasmic spaces (**Figure 7**). Because RNAP usually binds to DNA strongly, the dissociation of RNAP from the genomic DNA and hyper-condensed nucleoid only reported during osmotic stress (Cagliero and Jin, 2013). It is unlikely that the dissociations of RNAP under these two physiological conditions share common mechanisms, and understanding the molecular mechanism whereby RNAP dissociates from the nucleoid after chloramphenicol treatment is beyond the scope of this study. We speculate that the treatment of chloramphenicol causes significant changes in supercoiling states and/or the organization of the nucleoid, reducing the binding of RNAP released after completing transcription.

#### Active Transcription at Clusters of rRNA Operon Is Essential for the Formation of RNAP Foci and Nucleoid Compaction in the Mutants

**FIGURE 8:**
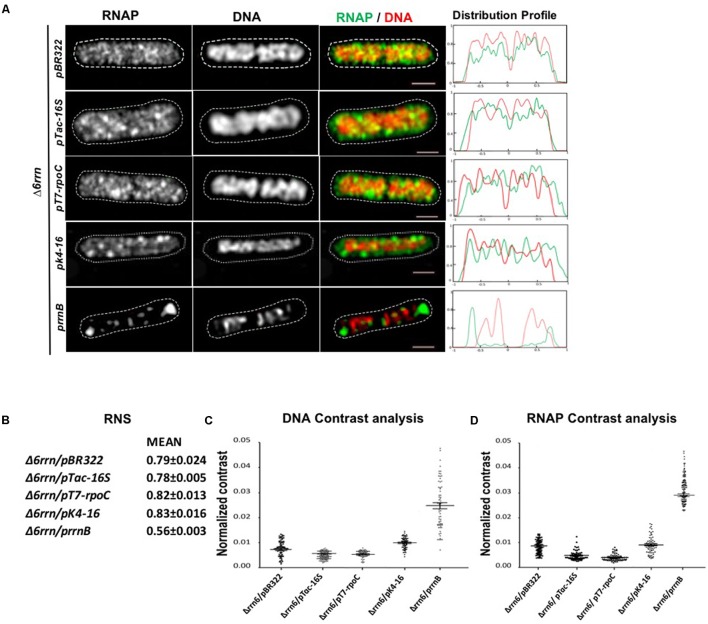
Active transcription at clusters of rRNA operon is essential for the compartmentalization of the extrachromosomal nucleolus-like organization and the nucleoid compaction. **(A)** Shown are SIM images of RNAP, DNA, and an overlay RNAP (green) and DNA (red) in representative single cells harboring different plasmids as indicated during optimal growth (LB, 37°C). Δ6*rrn/pBR322* and Δ6*rrn/*p*rrnB* are included as controls. RNAP is tagged with yellow Venus fluorescent protein (*rpoC*-venus) and DNA is stained with Hoechst 33342, as described in the Section “Materials and Methods.” Note that the defects in the Δ6*rrn* strain in the organization of RNAP foci and nucleoid structure cannot be complemented by a low copy plasmid-borne rrnB (*pK4-16*), nor by others two plasmids carrying strong promoters (pTac-16S and *pT7-rpoC*). The cell shape is outlined by a dotted line. The scale bar represents 1 μm. The two-color graphics show the distribution profiles of RNAP and nucleoid in a population of 100 cells, as described in the **Figure 1** caption. **(B–D)** Image analyses of the relative nucleoid size, nucleoid organization, and the distribution of RNAP. **(B)** Mean values of relative nucleoid size (RNS). **(C)** Normalized contrast parameters of the distributions of DNA. **(D)** Normalized contrast parameters of the distributions of RNAP signals in indicated strains. The normalized contrast parameters were measured from 100 cells as described in the Section “Materials and Methods” and in the **Figure 5** caption.

To determine whether active transcription from other strong promoters in multicopy plasmids also lead to RNAP foci formation, we examined the effects of pTac-16S and pT7-*rpoC* in Δ6*rrn* cells, and found that they behaved similarly to Δ6*rrn*/pBR322 cells in the distribution of RNAP and the nucleoid structure (**Figure 8**). To determine whether active transcription of *rrnB* from a low copy number plasmid has similar effects as multicopy p*rrnB*, we co-imaged RNAP and DNA in Δ6*rrn*/pK4-16. The plasmid pK4-16 contains *rrnB* in the pSC101 vector that has 3–4 copies in a log phase cell (Lutz and Bujard, 1997). The results showed in contrast to Δ6*rrn*/p*rrnB*, there are only small changes in the distribution of RNAP and the nucleoid structure in Δ6*rrn*/pK4-16 compared to Δ6*rrn*/pBR322 (**Figure 8**). From these results, we concluded that active transcription at clusters of rRNA operon is essential for the compartmentalization of the extrachromosomal nucleolus-like organization and the nucleoid compaction.

## Discussion

In this report, we determined the structure and function of the extrachromosomal bacterial nucleolus-like organization from plasmid-borne p*rrnB* in the Δ6*rrn*/p*rrnB* and Δ7*rrn*/p*rrnB* strains. Our study demonstrates that there are many similarities between RNAP foci at clusters of p*rrnB* and their counterparts at clusters of chromosomal rRNA operons. The results from the *rrn* deletion strains harboring p*rrnB* have implications for the formation of bacterial nucleolus-like organization from clustering of the rRNA operon in wild-type *E. coli* cells. This study also suggests that active transcription at transcription factories for rRNA synthesis and ribosome biogenesis is a nucleoid compaction force.

### The Transcription Machineries at Chromosomal *rrn* Clusters and at Extrachromosomal p*rrnB* Clusters Share Striking Similarities

There are many parallels in these two systems. First, they have similar compositions. In the Δ6*rrn*/p*rrnB* and Δ7*rrn*/p*rrnB* mutants, RNAP foci co-localize with NusB foci (**Figure 4A**) and function as transcription factories during optimal growth, just like in wild type. In addition to NusA and NusB, there may be other components (both protein and nucleic acids), because transcription factories are likely to be macro-structures containing multiple components. It is proposed that the organization of such macro-structures is entropy-driven (Marenduzzo et al., 2006). Putative transcription factories, which can be separated from the nucleoid, are evident from lysed cells of the two mutants (**Figure 6**). Thus, the mutant can be used as a useful system in the isolation and the identification of the components of transcription factories. Second, they have similar responses to environmental cues. The formation/organization of RNAP foci and p*rrnB* clusters are concurrent and colocalized under optimal growth conditions. However, in nutrient-poor minimal medium and stressed conditions, both RNAP and p*rrnB* are dispersed in the nucleoid (**Figure 3B**). Third, they are fully functional for rRNA synthesis and ribosome biogenesis. Note that in the Δ7*rrn*/p*rrnB* mutant, p*rrnB* is the only source for those functions to support cell growth; it is lethal for Δ7*rrn* in the absence of p*rrnB*.

In addition, transcription factories in both wild type and the Δ6*rrn*/p*rrnB* and Δ7*rrn*/p*rrnB* mutants are spatially compartmentalized at the edge of the nucleoid; however, they are predominantly located toward the cell poles in the mutants. We do not know why the cell poles are the preferred location for transcription factories in these mutants. Considering that the p*rrnB* clusters (on average 12 copies) are larger than the chromosomal *rrn* clusters (on average 6 copies), we speculate that there is more cytoplasmic space in the cell poles of these mutants that can accommodate the macro-structure of multiple components and avoid collision with the nucleoids. Larger chemoreceptor and other complexes are located in the cell poles (Maddock and Shapiro, 1993; Lybarger et al., 2005; Laloux and Jacobs-Wagner, 2014; Draper and Liphardt, 2017).

### Active Transcription Is Essential for rRNA Operons Clustering

Our results show that the formation and the organization of both RNAP foci and p*rrnB* clusters are sensitive to growth medium and conditions (**Figure 3B**). RNAP foci and p*rrnB* clusters are evident during optimal growth conditions; however, the distributions of both RNAP and p*rrnB* become random during nutrient downshift and stresses. We conclude from these results that active transcription of rRNA operons is essential for the clustering or assembling of p*rrnB*. In support of this conclusion, it has been reported that active transcription from a constitutive promoter is a driving force in assembling a relatively low copy plasmid (Sanchez-Romero et al., 2012). We further suggest that the same principle also applies to assembling and disassembling of chromosomal *rrn* clusters in response to growth medium and conditions in wild-type cells. Recently it was reported that colocalization of different chromosomal rRNA operons occurred with and without active transcription using epifluorescence microscopy and in living cells (Gaal et al., 2016). However, it has been demonstrated that live-cell imaging protocols, such as those used by Gaal et al. (2016), induce stress response and cause changes in the organization of the nucleoid (Cabrera and Jin, 2003; Jin et al., 2015). In addition, because *E. coli* cells are small it is essential to study the colocalization with superresolution microscopy. Thus, it remains to be determined whether different chromosomal rRNA operons are spatially in proximity under different growth conditions.

### Active Transcription at the Bacterial Nucleolus-Like Organization Is a Driving Force in Nucleoid Compaction?

In the absence of multicopy p*rrnB*, or in the presence of low copy plasmid-borne *rrnB*, the nucleoids are uncompacted in the Δ6*rrn* mutant host cells (**Figure 8**). However, when p*rrnB* is supplemented *in trans*, formations of RNAP foci and clusters of p*rrnB* condense the nucleoids in the Δ6*rrn*/p*rrnB* and Δ7*rrn*/p*rrnB* mutants during optimal growth conditions, demonstrating that active transcription at the extrachromosomal nucleolus-like organization is essential for nucleoid compaction. The structure of the nucleoid is proposed to be determined by a balance of expansion and compaction forces (Woldringh et al., 1995). Our study suggests that active transcription at extrachromosomal nucleolus-like organization is a nucleoid compaction force; however, how this force drives nucleoid compaction remains to be determined.

Because of the similarities between the organizations of transcription factories at extrachromosomal p*rrnB* clusters and at chromosomal *rrn* clusters, as detailed above, we argue that the two systems play a similar role in the condensation of the nucleoid in the cell. We speculate that active transcription of transcription factories not only for rRNA synthesis and ribosome biogenesis from clusters of rRNA operons but also for the expression of other growth genes at various locations in the genome through long-range interactions between bacterial nucleolus-like organization and the chromosome (Jin et al., 2017). RNAP is mobile (Bakshi et al., 2012; Endesfelder et al., 2013; Stracy et al., 2015) in transcription factories, and thus able to capture promoters of other growth genes in the genome. Active transcription of transcription factories induces supercoiling. DNA loops and supercoiling (Liu and Wang, 1987; Postow et al., 2004; Booker et al., 2010) could facilitate those long-range interactions, leading to nucleoid compaction. Recently, bacterial condensin has been shown to bind to multiple sites in rDNAs and other regions of the genome (Yano and Niki, 2017), which could provide another means for long-range interaction. In the future, identification and characterization of the components of transcription factories mentioned above will shed light on the mechanism of nucleoid compaction in the cell.

## Author Contributions

All authors designed the experiments, discussed the results, and contributed to the final manuscript. CM, ZS, and YZ performed the experiments and analyzed the data. CM and DJ wrote the manuscript.

## Conflict of Interest Statement

The authors declare that the research was conducted in the absence of any commercial or financial relationships that could be construed as a potential conflict of interest.
